# Using in silico methods to determine optimal tapering regimens for decanoate-based long-acting injectable psychosis drugs

**DOI:** 10.1177/20451253241272790

**Published:** 2024-09-12

**Authors:** James R. O’Neill, David M. Taylor, Mark A. Horowitz

**Affiliations:** Faculty of Medicine and Health, University of Leeds, LS2 9JT, UK; Maudsley Hospital, London, UK; Division of Psychiatry, University College London, London, UK

**Keywords:** antipsychotic, depot, discontinuation, flupentixol, haloperidol, hyperbolic, reduction, withdrawal, zuclopenthixol

## Abstract

**Background:**

Reducing the dose of psychosis drugs in a gradual hyperbolic manner may minimise withdrawal effects and risk of relapse. There is presently limited guidance on tapering decanoate-based long-acting injectable dopamine antagonists (LIDAs).

**Objectives:**

We aimed to apply hyperbolic principles of tapering to the decanoate-based LIDAs flupentixol, zuclopenthixol and haloperidol to develop withdrawal regimens.

**Design:**

We used in silico methodology to predict plasma drug levels and D_2_ occupancy for different LIDA regimens.

**Methods:**

Existing pharmacokinetic and receptor occupancy data from nuclear neuroimaging studies were used to power modelling. Abrupt discontinuation was examined as a potential strategy, and dose reduction was modelled with pre-defined constraints used in similar work of 10 (fast regimens), 5 (moderate) and 2.5 (slow) percentage points of D_2_ occupancy change per month.

**Results:**

Abrupt discontinuation of decanoate-based LIDAs leads to excessive change in D_2_ occupancy which violated our pre-defined constraints, potentially resulting in withdrawal symptoms and increased risk of relapse. Reduction of LIDA dose allowed hyperbolic reduction in plasma level consistent with imposed constraints on receptor occupancy reduction rate. For equivalent per-weekly LIDA dosing, more frequent administration allowed a more gradual reduction of D_2_ occupancy. However, switching to oral forms is required to continue hyperbolic tapering to full discontinuation; reduction to zero using only LIDA produces too large a reduction in D_2_ occupancy. Guidance for reduction and cessation of LIDAs according to slow, moderate and fast criteria is provided.

**Conclusion:**

Abrupt cessation of decanoate LIDAs is not consistent with gradual hyperbolic tapering, despite their longer half-lives compared with oral formulations. Reduction to the point of full discontinuation can only be achieved by switching to oral therapy to complete the taper. These results are limited by the in silico and theoretical nature of the study, and there is a need to confirm these findings through real-world observational and interventional studies.

## Plain language summary

Computer-based research on the best way to reduce the dose and eventually stop three depot antipsychotics

What is the problem?

Psychosis affects how someone experiences reality. Antipsychotics are used to treat psychosis. They work by blocking a chemical called dopamine. Stopping these too rapidly can cause psychosis to occur again. This might be because the dopamine block is removed suddenly. Sometimes antipsychotics are given by injection. Injections stay in the body for a longer time than tablets. The best way to reduce these injections is currently unknown.

What did we do?

We explored the best way to reduce and stop antipsychotic injections. We looked at three different types of psychosis injection. We used existing data to assess how these drugs affect the brain. We examined what would happen if an antipsychotic injection was suddenly stopped. We then evaluated the effect of reducing the dose gradually.

What did we find?

Stopping antipsychotic injections suddenly would lead to rapid changes at brain receptors. This might lead to symptoms of withdrawal and a high risk of psychosis recurring. Reducing the dose of injections leads to less change than stopping suddenly. Switching to a tablet allows for more control when reducing dose. Having injections more often also reduce the changes occurring between injections. We outline three different rates of dose reduction.

What does this mean?

Antipsychotic injections stay in the body longer than tablets. However, stopping injections suddenly may still not be safe. We recommend that people reduce the dose of their injection instead. We also recommend people switch to tablets or liquid before stopping. It should be noted that our research is computer-based. We would like to see our recommendations tested in the real world.

## Background

There were over 70,000 prescriptions for decanoate-based long-acting injectable dopamine antagonists (LIDAs), also known as ‘antipsychotic depots’, within primary care alone during the past year,^1^ with the data not accounting for those prescribed in secondary care or elsewhere. Flupentixol decanoate and zuclopenthixol decanoate are the most and second-most prescribed long-acting psychosis drugs in the UK,^2^ with haloperidol decanoate also being commonly prescribed.

Although a recent trial found no short- or moderate-term improvements with reduction or discontinuation of psychosis drugs,^3^ it has been suggested that discontinuing antipsychotics may improve long-term prognosis through improving functional remission and increasing the likelihood of recovery for some patients.^4,5^ Up to 40% of people with psychotic conditions may be able to achieve good long-term outcomes without maintenance pharmacological treatment.^6^ In addition, qualitative data^7^ from the aforementioned trial^3^ details how participants experienced both improvements in functioning as well as withdrawal symptoms, suggesting that dose tapering during the trial may have taken place too quickly, and benefits accrued to a sub-set of patients.

While abrupt discontinuation of psychotic drugs is likely to lead to relapse of psychotic symptoms,^8,9^ this risk of relapse is thought to be reduced when a more gradual approach is taken with dose tapering.^10–12^ Reducing dosing in a hyperbolic manner, resulting in linear reductions in both D_2_ receptor occupancy^13^ and symptom scores,^14,15^ may limit the emergence of withdrawal symptoms and reduce the risk of relapse.^16–21^

Previously, it has been suggested that tapering regimens using oral medications corresponding to 2.5, 5 and 10 percentage point reduction per month (consistent with tapering over approximately 9, 18 and 36 months) may represent tolerable reduction schedules for patients on dopamine antagonists.^16^ There is some empirical support for this,^5,22^ although these ideas require further testing.^19,23^ It may be that some patients require even slower tapering than 2.5 percentage point reductions per month,^5^ so tapering regimens must be individualised.

Tapering in long-acting form may be beneficial for patients who have adverse effects from their medication. LIDAs offer clinical utility by avoiding daily medication burden, as well as improving concordance^24^ during both maintenance treatment and potential reduction regimens. However, limited research and guidance currently exist on the rate and individual steps required to reduce and discontinue psychosis drugs while avoiding destabilisation of the underlying condition, especially with regard to LIDAs.

The extent of D_2_ occupancy change that can be tolerated during inter-dose intervals between LIDA administrations remains unclear. Observational studies are currently investigating fluctuations in mental state and plasma levels during LIDA inter-dose intervals.^25^ We have previously demonstrated that psychosis drugs with longer half-lives may be tapered through extending the inter-dose interval, which results in a lower steady-state range.^26^ However, decanoate-based LIDAs have shorter half-lives. This may lead to a greater rate of D_2_ occupancy change (RODOC) during their inter-dose intervals, making tapering through extension of the inter-dose interval less feasible.

Hyperbolic tapering often requires the use of minute doses of medication prior to full discontinuation.^16^ Decanoate-based LIDAs are drawn up individually from vials of medication,^27^ allowing administration of small and precise quantities that may allow for such a regimen. However, as the dose of LIDA is reduced, the RODOC during the inter-dose interval will increase,^26^ leading to significant fluctuation in D_2_ occupancy during inter-dose intervals. This would be particularly pronounced at minute doses often recommended for hyperbolic tapering prior to discontinuation.^16^ Therefore, D_2_ occupancy change during inter-dose intervals may be a more appropriate indicator for tapering thresholds.^25^

## Objectives

Our overall aim was to determine regimens for reducing decanoate-based LIDAs that were consistent with maximum RODOC constraints of 2.5, 5 and 10 percentage point reduction per month (consistent with point reductions of oral tapering regimens aiming for discontinuation over approximately 9, 18 and 36 months), which have been previously suggested.^16^

We first determined whether the D_2_ occupancy change per month resulting from abrupt discontinuation of each of three decanoate-based LIDAs^28–30^ was consistent with these constraints.

We then aimed to determine D_2_ occupancy change during the inter-dose interval of various doses of LIDA medications, in order to determine whether these could be tapered in a hyperbolic manner similar to that previously proposed for oral medications.^16^

Finally, we aimed to devise dose reduction regimens that would fit the pre-determined constraints of RODOC. Where it was not possible to continue a LIDA taper to full discontinuation within these thresholds, we aimed to determine an appropriate oral switch option for tapering to be continued.

## Methods

### LIDA dose-plasma concentration data

Half-life^31^ and *t*_max_^32–34^ information for the three decanoate-based LIDAs have been taken from existing literature, and pharmacokinetic studies for flupentixol decanoate,^35–37^ haloperidol decanoate^33^ and zuclopenthixol decanoate^32^ were used to devise in silico models for plasma levels over time. Equations used to calculate pharmacokinetic modelling for inter-dose intervals of the three LIDAs are outlined further in Supplemental Table 1.

### Oral dose-plasma concentration data

Dose–concentration ratios were determined for oral haloperidol using previous literature.^38^ The correlation between dose and plasma concentration has been determined to be linear; a 10 mg dose corresponded to plasma level of 28.22 nmol/l, which was converted to 10.61 ng/ml using haloperidol’s molar mass of 375.9 g/mol.^39^

Flupentixol plasma levels have also been found to linearly correlate with oral dose.^40^ References of a 1.5 mg dose resulting in a maximal plasma concentration of 0.495 ng/ml,^41^ as well as 1 mg dosing corresponding to a trough level of 0.2 ng/ml,^42^ were used to extrapolate further results which correlated approximately to other resultant dose–concentration findings.^43^

The product monograph for Clopixol^©^ (zuclopenthixol dihydrochloride) tablets states that a 20 mg oral dose corresponds to a plasma level of 13 ng/ml, and the dose–concentration ratio is again understood to be linear in nature.^42^

### D_2_ occupancy data

The Michaelis–Menten equation for receptor occupancy is:

$$\mathrm{Occupancy}\left(\%\right)\;=\;{Ε}_{\max}*\mathrm{PC}/\;\left(\mathrm{PC}+{\mathrm{EC}}_{50}\right),$$

where PC is plasma concentration, *E*_max_ is the determined maximal occupancy of receptors and EC_50_ is the plasma drug concentration required to generate 50% of maximal occupancy.^13,43,44^ Characteristics for the three decanoate-based D_2_ antagonists were derived from previous literature utilising striatal neuroimaging using [^11^C]-raclopride binding to unoccupied receptors.^43–45^ Each of these imaging studies assumed an *E*_max_ value of 100% for each drug. EC_50_ values were given as 0.68 ng/ml for flupentixol^43^ and 0.51 ng/ml for haloperidol.^45^ The EC_50_ value for zuclopenthixol was determined to be 2.887 nmol/L,^44^ which was converted to 1.158 ng/ml using zuclopenthixol’s molar mass of 400.965 g/mol.^46^

### Plasma concentration modelling

Plasma concentrations were modelled in silico using Microsoft Excel software (version 2209, 64-bit). Models were based on hypothetical patients already established on LIDA having achieved steady-state plasma levels at a certain dose and administration frequency. Alterations were made to dosing to observe the impact on both plasma level and D_2_ occupancy. Remaining proportions of drug were calculated at various intervals using a free-to-use online half-life calculator.^47^

### Determining LIDA regimens

Absolute change in D_2_ occupancy during the inter-dose interval, or over 30 days for abrupt discontinuation, was used to guide RODOC thresholds for tapering regimens, equating to 2.5 (slow), 5 (moderate) and 10 (fast) percentage points.

Dosing regimens for LIDA were rounded up from the threshold value to the nearest 0.2 ml of the current lowest available concentration of decanoate LIDA for ease of administration. This corresponded to incremental differences of 4 mg (doses less than 60 mg) or 20 mg (doses greater than 60 mg) for flupentixol,^30,48^ 40 mg for zuclopenthixol^29^, and 10 mg (doses less than 150 mg) or 20 mg (doses greater than 150 mg) for haloperidol.^28,48^

## Results

### Abrupt discontinuation from maximum licensed LIDA dose

We first examined the effect of abrupt discontinuation of the maximum licensed doses of three decanoate-based LIDAs. Values for peak plasma concentration (*C*_max_) were calculated, and are shown in Table 1, along with other key pharmacokinetic characteristics.

**Table 1.** Pharmacokinetic and pharmacodynamic information for the three LIDAs modelled in our study at their maximum licensed doses.

Drug	Half-life (days)	*t*_max_ (days)	Maximum licensed dose^28–30^	*C*_max_ (ng/ml)	*C*_max_ D_2_ occupancy (%)^43–45^
Flupentixol decanoate^35–37^	17^34^	7^34^	400 mg weekly	102.61	99.34
Haloperidol decanoate^33^	20^33^	7^33^	300 mg 4-weekly	9.65	94.98
Zuclopenthixol decanoate^32^	19^31^	7^32^	600 mg weekly	45.73	97.53

LIDA, long-acting injectable dopamine antagonist.

**Figure 1.**
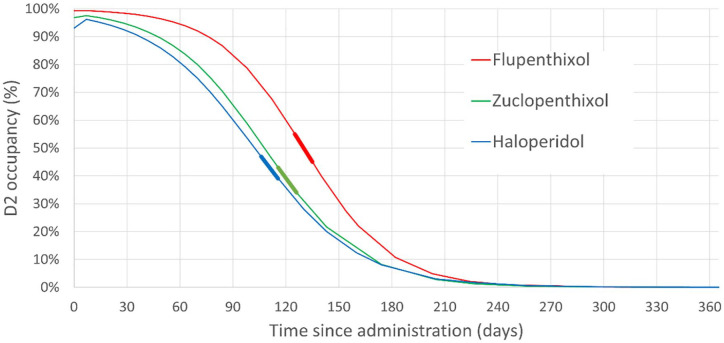
Graph showing the change in D_2_ occupancy resulting from abrupt discontinuation of three decanoate-based LIDAs. Bold lines of corresponding colour demonstrate the peak RODOC for each LIDA. LIDA, long-acting injectable dopamine antagonist.

Figure 1 demonstrates the RODOC resulting from abrupt discontinuation of the three LIDAs. Peak RODOC (%/30 days) was observed to be 27.47 for flupentixol, 26.63 for zuclopenthixol and 24.86 for haloperidol. Each of these values far exceeded the pre-defined highest acceptable threshold of 10 percentage points per 30 days.

Modelled values for peak RODOC are dependent upon the half-life of the drug, rather than dosage. Therefore, peak RODOC values will be the same irrespective of dose. However, while periods of maximal RODOC occurred several months after cessation of these maximum licensing doses, this will manifest earlier at lower doses. Effects of abrupt discontinuation from the ‘minimum effective dose’^49^ are outlined in Supplemental Table 2 and Supplemental Figure 1, showing the same maximal RODOC values as for maximum licensed doses.

### D_2_ occupancy change during inter-dose intervals

Six different example doses and administration frequencies for flupentixol decanoate have been outlined for demonstration purposes in Table 2. Table 2(a) outlines the effect of different frequencies of administration of the same dosage, while Table 2(b) outlines three flupentixol decanoate regimens of equal per-weekly dosing but varying administration frequency. These examples demonstrate that longer inter-dose intervals result in greater peak-to-trough ratio in plasma level, as well as greater RODOC, despite the total dose delivered being the same. Further examples of haloperidol and zuclopenthixol decanoate are shown in Supplemental Table 3.

**Table 2.** Tables showing modelled pharmacokinetics and respective D_2_ occupancies for various regimens of flupentixol decanoate.

Flupentixol dose and frequency	*C*_min_ (ng/ml)	D_2_ occupancy at *C*_min_ (%)	*C*_max_ (ng/ml)	D_2_ occupancy at *C*_max_ (%)	Absolute D_2_ occ. change during inter-dose interval (percentage points)
(a)					
200 mg 2-weekly	21.62	96.95	29.69	97.76	0.81
200 mg 3-weekly	10.48	93.91	18.55	96.46	2.56
200 mg 4-weekly	5.96	89.75	14.03	95.38	5.63
(b)					
80 mg 2-weekly	8.65	92.71	11.87	94.58	1.87
120 mg 3-weekly	6.29	90.24	11.13	94.24	4.00
160 mg 4-weekly	4.77	87.51	11.22	94.29	6.78

### Thresholds for LIDA tapering

Table 3 outlines the thresholds for LIDA tapering for three differing speeds of taper, showing the lowest dosages that regimens can reduce before excessive change in D_2_ occupancy is observed during the inter-dose interval. This shows dosages rounded up to the next 0.2 ml dosing increment to allow for practical administration in a clinical setting, but dosages to the closest milligram can be found in Supplemental Table 4. Any modelled dosages higher than the maximum licensed dose have not been included.

**Table 3.** Thresholds for breaching regimen-specific D_2_ occupancy change for three LIDAs, in terms of dose in milligrams rounded up to the next 0.2 ml dosing increment currently available.^28–30^

Drug	Slow	Moderate	Fast
Flupentixol	60 mg 2-weekly 220 mg 3-weekly N/A 4 weekly	24 mg 2-weekly 100 mg 3-weekly 240 mg 4-weekly	8 mg 2-weekly 36 mg 3-weekly 100 mg 4-weekly
Haloperidol	90 mg 2-weekly N/A 3-weekly N/A 4-weekly	40 mg 2-weekly 180 mg 3-weekly N/A 4-weekly	10 mg 2-weekly 70 mg 3-weekly 200 mg 4-weekly
Zuclopenthixol	200 mg 2-weekly N/A 3-weekly N/A 4-weekly	80 mg 2-weekly 400 mg 3-weekly N/A 4-weekly	40 mg 2-weekly 160 mg 3-weekly 400 mg 4-weekly

LIDA, long-acting injectable dopamine antagonists; N/A = no dosing below the licensed maximum at this interval will result in D_2_ occupancy change within the defined tapering threshold. This means it is not possible to taper LIDA at this frequency of administration in such a manner that will be consistent with gradual, hyperbolic tapering.

Each of these thresholds demonstrates the lowest dosage that can be reduced to in LIDA form, resulting in absolute difference of no greater than 2.5 (for slow regimens), 5 (for moderate regimens) or 10 (for fast regimens) D_2_ occupancy percentage point reduction during the inter-dose interval. It has been noted that tapering regimens may need to progress at an even slower rate, and a further ‘very slow’ regimen of 1.25 percentage point change during inter-dose interval is also outlined in Supplemental Table 4.

Due to the large change in RODOC when LIDAs are administered 3-weekly and 4-weekly, there are numerous instances where tapering at these intervals is not possible to conduct in a gradual, hyperbolic manner. In such instances, LIDAs will need to be administered more frequently or switched to oral medication.

Regimens may also be defined in terms of ‘trough’ plasma concentration (*C*_min_). This would eliminate interpersonal variation in response to certain dosages,^34^ and therapeutic drug monitoring may therefore allow for more accuracy in prescribing tapering regimens.^50^ *C*_min_ thresholds are outlined in Supplemental Table 5.

### Implementing thresholds to determine dosing regimens

Reduction to the threshold LIDA dose can be made without stepwise reduction in dosage due to progressive elimination of the previous higher dose. This would result in hyperbolic reduction in plasma level, and therefore linear reduction in D_2_ occupancy.

**Figure 2.**
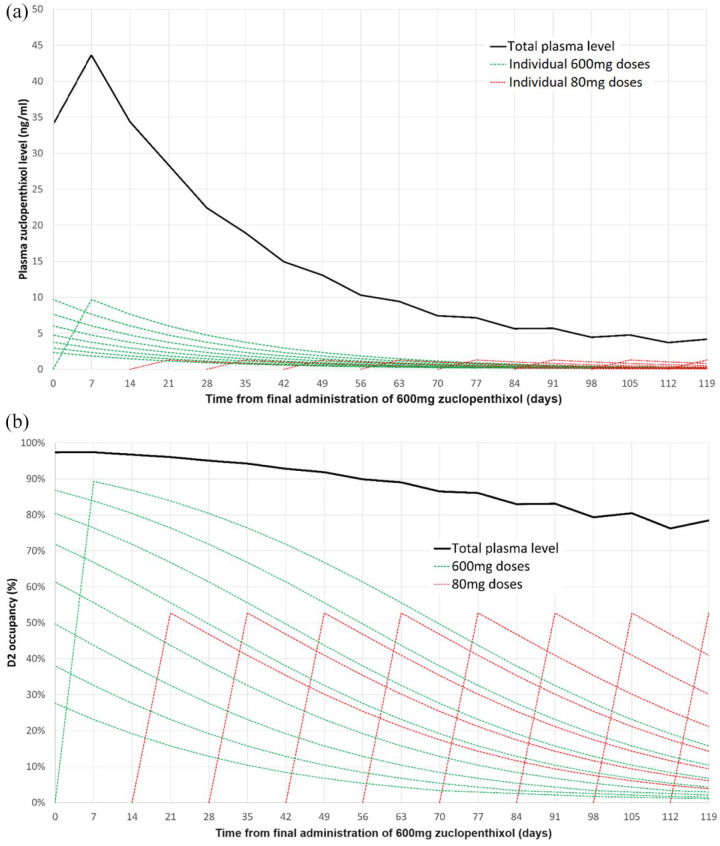
Graph showing progressive change in (a) plasma level and (b) D_2_ occupancy resulting from dose reduction from 600 mg weekly, to 2-weekly doses of 80 mg for zuclopenthixol decanoate, following our moderate tapering regimen. Green dashed line shows residual zuclopenthixol plasma levels from individual doses given at *t* ⩽ 0. Red dashed lines show individual 80 mg doses given fortnightly. As can be seen from the graphs, plasma levels and D_2_ occupancy will continue to drop for months after the transition as the original LIDA is eliminated, before reaching a new lower steady state. LIDA, long-acting injectable dopamine antagonists.

For example, when a patient established on a dose of 600 mg zuclopenthixol weekly follows the moderate tapering schedule outlined in Table 3, dose may be reduced to 80 mg fortnightly without intermediate dosing. The new reduced dosing should be continued for at least 2–3 months in order to allow for steady state to be reached while the previous LIDA dose is fully eliminated. This is demonstrated in Figure 2.

The full effects of dose reduction cannot be adequately assessed until plasma levels reach a steady state of *C*_max_ and *C*_min_ values, which typically take around four half-lives to be achieved.^51^ Therefore, further reductions should not take place at least until steady-state levels for the new dosing regimen have been reached, which would take around 3 months for decanoate-based LIDAs. If even slower reduction schedules are required, for example, 1.25 percentage point decreases per month, a higher threshold LIDA dose would be required before switching to oral medication to continue implementing dose tapering.

### Oral switch for continuing discontinuation

**Figure 3.**
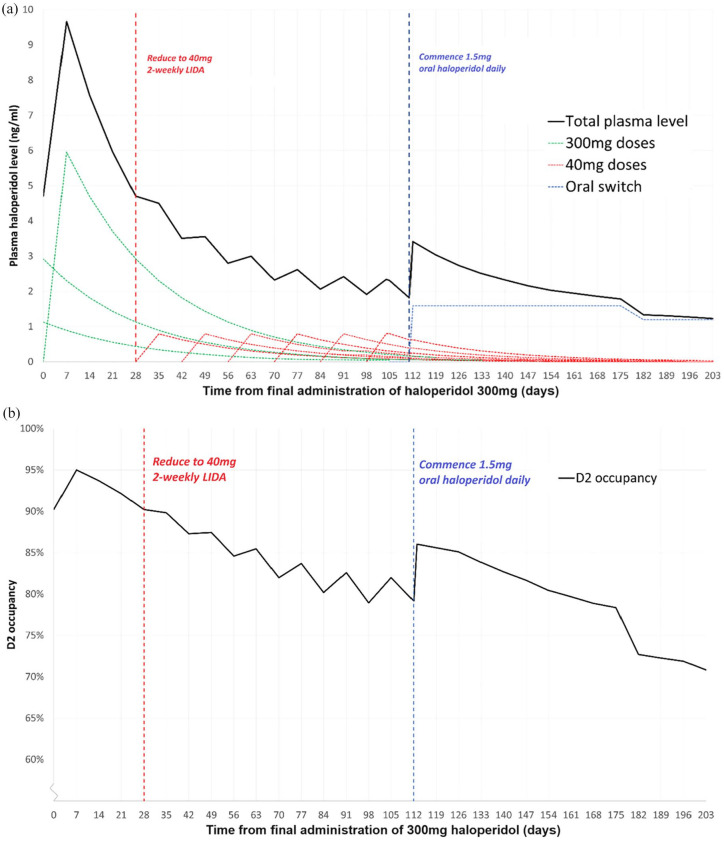
Graph showing the moderate tapering regimen for haloperidol, as outlined in Table 4, in terms of (a) plasma haloperidol level and (b) D_2_ occupancy. For (a), solid black line represents total plasma level of haloperidol. Dashed lines show individual effects of original haloperidol decanoate dose (green), tapered haloperidol decanoate dose (red) and oral switch (blue).

Tapering below the dosages listed in Table 3 will require switching to oral forms in order to maintain consistency with the pre-defined RODOC constraints. Oral forms are dosed more frequently and allow greater control over changes in plasma level, and therefore D_2_ occupancy. As we have previously demonstrated for aripiprazole,^26^ switching to oral medication in order to continue discontinuation should account for residual LIDA that is eliminated concurrently. Table 4 outlines appropriate tapering regimens including recommendations for oral switching, while Figure 3 demonstrates in graphical form a moderate taper of haloperidol from maximum licensing dose of 300 mg 4-weekly.

**Table 4.** Recommended oral switch regimens in order to continue discontinuation past threshold LIDA dosing.

Drug	Slow	Moderate	Fast
Flupentixol	- Starting LIDA dose - Reduce to 60 mg 2-weekly LIDA - Maintain dose and frequency for 3 months - When next LIDA dose due, commence 13 mg oral flupentixol daily. - Maintain dose for 2 months. - Continue monthly reductions at a rate consistent with 2.5 percentage points per month.	- Starting LIDA dose - Reduce to 24 mg 2-weekly LIDA - Maintain dose and frequency for 3 months - When next LIDA dose due, commence 7.5 mg oral flupentixol daily. - Maintain dose for 2 months. - Continue monthly reductions at a rate consistent with 5 percentage points per month.	- Starting LIDA dose - Reduce to 8 mg 2-weekly LIDA - Maintain dose and frequency for 3 months - When next LIDA dose due, commence 3 mg oral flupentixol daily. - Maintain dose for 2 months. - Continue monthly reductions at a rate consistent with 10 percentage points per month.
Haloperidol	- Starting LIDA dose - Reduce to 90 mg 2-weekly LIDA. - Maintain dose and frequency for 3 months - When next LIDA dose due, commence 2 mg oral haloperidol daily. - Maintain dose for 2 months. - Continue monthly reductions at a rate consistent with 2.5 percentage points per month^16^	- Starting LIDA dose- Reduce to 40 mg 2-weekly LIDA. - Maintain dose and frequency for 3 months - When next LIDA dose due, commence 1.5 mg oral haloperidol daily. - Maintain dose for 2 months. - Continue monthly reductions at a rate consistent with 5 percentage points per month^16^	- Starting LIDA dose - Reduce to 10 mg 2-weekly LIDA. - Maintain dose and frequency for 3 months - When next LIDA dose due, commence 500 MICROgrams oral haloperidol daily. - Maintain dose for 2 months. - Continue monthly reductions at a rate consistent with 10 percentage points per month^16^
Zuclopenthixol	- Starting LIDA dose. - Reduce to 200 mg 2-weekly LIDA - Maintain dose and frequency for 3 months - When next LIDA dose due, commence 8 mg oral zuclopenthixol daily. - Maintain dose for 2 months. - Continue monthly reductions at a rate consistent with 2.5 percentage points per month.	- Starting LIDA dose. - Reduce to 80 mg 2-weekly LIDA - Maintain dose and frequency for 3 months - When next LIDA dose due, commence 5 mg oral zuclopenthixol daily. - Maintain dose for 2 months. - Continue monthly reductions at a rate consistent with 5 percentage points per month.	- Starting LIDA dose. - Reduce to 40 mg 2-weekly LIDA - Maintain dose and frequency for 3 months - When next LIDA dose due, commence 2 mg oral zuclopenthixol daily. - Maintain dose for 2 months. - Continue monthly reductions at a rate consistent with 10 percentage points per month.

LIDA, long-acting injectable dopamine antagonists.

## Discussion

Our in silico modelling for decanoate-based LIDAs demonstrates that abrupt cessation would lead to excessively rapid RODOC which would violate our pre-defined maximum constraints of 10 percentage points of D_2_ occupancy change during the inter-dose interval. Abrupt discontinuation might therefore increase the risk of withdrawal effects or risk of relapse.

Tapering of decanoate LIDAs can be implemented with a single reduction in dose, rather than in a stepwise fashion, due to the prolonged half-life causing slowed elimination of previously administered LIDA doses. This would result in a gradual reduction in plasma drug level which would be consistent with pre-defined constraints of RODOC. However, switching to oral forms is required to complete a tapering regimen to the point of full discontinuation, due to the greater degree of control that more frequent dosing allows. We have developed dose reduction regimens that could be implemented in clinical practice when LIDA dose reduction is indicated.

### Strengths and limitations

One strength of this work is that it is based on real-world pharmacokinetic and neuroimaging data. Although pharmacokinetic and neuroimaging data for zuclopenthixol decanoate was exclusively conducted on males,^32,44^ there was an equal split in terms of sex for flupentixol in terms of both pharmacokinetic^36^ and neuroimaging^43^ data. This pharmacokinetic data was then used to extrapolate dose–concentration relationships and elimination half-lives.

However, the main limitation of this approach is the use of in silico methodology to predict and anticipate effects of reducing dosage. Recommendations outlined here remain theoretical until they can be clarified by real-world observation of patients reducing and discontinuing antipsychotics, with studies around the world currently ongoing.^23,25^ In addition, prediction of plasma levels utilises the mean resultant plasma concentrations for certain doses, which may not apply to all patients.

Significant interpersonal variation exists for dose–concentration relationships. Flupentixol decanoate half-life, for example, has been observed to vary six-fold between different patients.^34^ This limits the potential for universal application of these guidelines to all patients, and determining an individual’s metaboliser status^52^ or plasma concentration on existing dosage^50^ may be useful in order to personalise tapering regimens, ensuring that intended RODOC is not breached unwittingly.

This underlines the importance of clinical acumen throughout the tapering process. Ultimately, a patient’s clinical response to dose reduction is more important than strict adherence to any suggested schedule. Evidence of deterioration would be reason for halting dose reductions, returning to a dose that has previously resulted in stable mental state, followed by reductions at a much more gradual rate.^12^ Therefore, regimens developed here, as with any tapering schedule, should only be seen as a guideline around which variations can be made to suit the individual. Some patients may require even slower reductions than are outlined here.

### Abrupt discontinuation of LIDA

Stopping decanoate-based LIDA medication abruptly may reduce risk of relapse when compared to stopping oral forms,^11^ but modelling suggests this would still produce a more rapid reduction in D_2_ occupancy than has been suggested to minimise risk of relapse.^20,26^ Abrupt cessation of decanoate LIDAs should therefore not be considered a suitable tapering strategy for implementing a gradual hyperbolic taper.

Time to peak RODOC following abrupt discontinuation will vary depending on drug and dose. For maximal licensing doses of LIDA medications, this will occur at around 4 months following discontinuation, but lower doses will result in the peak RODOC being observed earlier than our modelling suggests. This provides one reason why relapses following LIDA cessation may be delayed for some time.

It is noted that all three LIDAs have maximum licensing doses which appear to typically exceed 95% D_2_ receptor occupancy. It has been suggested that exceeding 80% D_2_ occupancy both increases the risk of cognitive impairment, as well as causes extra-pyramidal side effects.^53^ Clinicians might therefore consider dose reduction in a measured and hyperbolic manner for patients on high doses of these medications.^54^

### LIDA tapering regimens

We have outlined potential differences between the tapering of long-acting injectable and oral medications that prescribers should account for when attempting to deprescribe psychosis drugs. We found that tapering of high-dose LIDA medication may be possible to certain doses, but steeper reductions in D_2_ occupancy occur as dose reduces below recommended thresholds.

This is particularly the case for long-acting psychosis drugs administered with longer inter-dose intervals, which result in a greater peak-to-trough ratio and subsequently higher RODOC. Therefore, varying frequency of administration will not result in like-for-like RODOC for the same per-weekly dosing, and more frequent dosing would lead to reduced RODOC. Tapering of dose may therefore be more tolerable with more frequent administration of dose. For further reductions below these threshold doses, switching to oral forms may be required.

### Rate versus absolute change in D_2_ occupancy as outcome measure

For previous in silico analysis of long-acting psychosis drugs,^26^ RODOC during the inter-dose interval has been defined in terms of the ‘maximal’ (highest RODOC over a period of 7 days) rate, rather than over the course of a whole month. We based these constraints on the same suggested thresholds used for oral tapering regimens.^16^ However, these were adjusted to account for a more gradual reduction in plasma level with LIDAs over the course of weeks due to their longer half-life (compared with the 24 h for oral medication), aiming for equivalence with oral forms in terms of tolerability. As a result, maximal RODOC thresholds of 5 (slow), 7.5. (moderate) and 12.5 (fast) percentage points over 30 days were used.

However, the shorter half-lives, and therefore more rapid RODOC, of decanoate-based LIDAs means that a similar approach is not as justifiable as for long-acting psychosis drugs with longer half-lives such as aripiprazole monohydrate.^26^ As a result, it was decided to focus instead on absolute change observed during the inter-dose interval, or for abrupt discontinuation over a 30-day period. This is more aligned with proposed constraints used for oral tapering thresholds.^16^ It is clear that further research is required in terms of the most accurate measure and definition of tapering thresholds, including observational study of long-acting psychosis drugs^25^ and randomised controlled trials of the proposed reduction and discontinuation strategies.

### Rates of reduction and discontinuation

We hypothesise that a tapering regimen producing reduced RODOC would lead to lessened impact of dose reduction, limiting the potential impact from dopamine antagonist withdrawal and, in turn, effects on a person’s mental state.

A recent study has suggested that the discontinuation of paliperidone in any medicinal form, whether oral, 1-monthly long-acting or 3-monthly long-acting, does not produce any difference in relapse rates.^55^ However, comparisons used as control groups in this study for oral and long-acting medication may not be like-for-like, due to greater adherence to long-acting injectable medication than with oral forms. Comparing absolute relapse rates between oral, 1-monthly (1MPP) and 3-monthly paliperidone palmitate (3MPP) shows a smaller overall rate of relapse for 3MPP compared to oral medication at a time point that allows for elimination of the 3MPP.^56^ This is consistent with the findings of other studies.^11^

Moreover, discontinuation from steady state of 3MPP and its modified transport substrate^57,58^ was not assessed, given that the only 3MPP trial^59^ included only administered this for one dose, instead of the 4–5 administrations required to establish steady state.^51^

The current rate at which psychosis drugs can be safely discontinued remains unclear. Theoretical threshold RODOC values have been suggested, but these still require real-world testing. Although a trial^3^ has recently found increased rates of relapse and significant incidents with discontinuation, this may be because this study adopted a linear approach to dose reduction over a period of 12–18 months,^60^ which may have produced excessive RODOC for participants.

Another trial assessed the impact of guided dose reduction to minimum effective dosing against maintenance therapy,^5^ rather than full discontinuation. Rates of relapse were not different between dose reduction and maintenance groups with hyperbolic reductions of no more than 25% at a time allowing 6 months between reductions. This would equate roughly to point D_2_ occupancy reductions of around 2–2.5 percentage points, and a per-monthly RODOC of approximately 0.3–0.4 percentage points. This allowed almost three-quarters of patients in the reduction arm to be able to reduce their dose while remaining in remission.^5^

## Conclusion

In conclusion, we present tapering regimens for some of the most prescribed LIDAs in the UK.^2^ We outline that abrupt discontinuation will produce a more rapid rate of D_2_ occupancy decline than anticipated, increasing the risk of withdrawal symptoms or destabilisation leading to relapse.

Instead, reduction of LIDA dose should take place cautiously on an individual case-by-case basis to lower dosage, using the guidelines we have produced as a framework, before switching to oral forms for continued tapering to complete discontinuation. The presence of residual previously administered LIDA, both when reducing to a lower injectable dose and when switching to an oral formulation, will ‘smooth’ the decline in plasma levels and D_2_ occupancy, which may make the process less disruptive for the patient. These residual effects are accounted for in the regimens presented.

Careful dose tapering according to these principles may reduce the adverse effect burden for patients, and potentially improve their functional status, without destabilising their mental health conditions. As these guidelines derive from in silico calculations based on population averages, these regimens require testing in a real-world randomised controlled trial to clarify understanding around effective reduction and discontinuation of psychotropic medications.

## Supplemental Material

sj-docx-1-tpp-10.1177_20451253241272790 – Supplemental material for Using in silico methods to determine optimal tapering regimens for decanoate-based long-acting injectable psychosis drugs Supplemental material, sj-docx-1-tpp-10.1177_20451253241272790 for Using in silico methods to determine optimal tapering regimens for decanoate-based long-acting injectable psychosis drugs by James R. O’Neill, David M. Taylor and Mark A. Horowitz in Therapeutic Advances in Psychopharmacology

## Declarations

**Ethics approval and consent to participate:** This project did not involve any original participants, and as such did not require ethical approval.

**Consent for publication:** This project did not involve any original participants, and as such consent for publication is not applicable nor required.

**Competing interests:** J.O. is a member of the Tapering Antipsychotics and Evaluating Recovery (TAPER) group consisting of international psychiatric researchers. M.A.H. is Associate Editor of Therapeutic Advances in Psychopharmacology. He is also the Clinical Research Fellow on the Research into Antipsychotic Discontinuation And Reduction (RADAR) study, which is funded by the National Institute for Health Research (NIHR). He is an unpaid associate of the International Institute of Psychiatric Drug Withdrawal and a member of the Tapering Antipsychotics and Evaluating Recovery (TAPER) group consisting of international psychiatric researchers. D.M.T. is the Editor-in-Chief of Therapeutic Advances in Psychopharmacology. He is a shareholder in Myogenes and 428 Pharma. He has received research funding from Janssen Pharmaceuticals and Lundbeck, but no sources of funding were received for the preparation of this article. He has also received speaking honoraria from Janssen, Otsuka, Viatris, Lundbeck, Sunovion and Recordati. As the authors of this paper include the Editor-in-Chief and Associate Editor of Therapeutic Advances in Psychopharmacology, the peer-review process was managed by alternative members of the board and the submitting editors have not been involved in the decision-making process.

**Availability of data and materials:** Further modelling data or methodology used in this study can be requested from the lead author.
