# Supplementary material for: Using in silico methods to determine optimal tapering regimens for decanoate-based long-acting injectable psychosis drugs
Source: Ther Adv Psychopharmacol. 2024 Sep 12;14:20451253241272790. doi: 10.1177/20451253241272790 (PMC11401015; doi:10.1177/20451253241272790)
Supplement: sj-docx-1-tpp-10.1177_20451253241272790 – Supplemental material for Using in silico methods to determine optimal tapering regimens for decanoate-based long-acting injectable psychosis drugs [file sj-docx-1-tpp-10.1177_20451253241272790.docx]

**SUPPLEMENTARY MATERIALS**

**Supplementary Table 1**

|  | **C_min_ (ng/ml)** | **C_diff_ (ng/ml)** |
| --- | --- | --- |
| **Flupentixol** | (for 2-weekly) 0.10809 * dose in mg | 0.04034 * dose in mg |
| **Haloperidol** | (for 3-weekly) 0.02284 * dose in mg | 0.01984 * dose in mg |
| **Zuclopenthixol** | (for 3-weekly) 0.01314 * dose in mg | 0.01846 * dose in mg |

*Table outlining equations used for in silico pharmacokinetic modelling.
C_diff_ = C_max_ – C_min_
Methodology for these equations is as follows:
Flupentixol – Pharmacokinetic data* (33–35) *was extracted for C_min_ from 2-weekly dosing regimens, and a least-squares regression method using a zero intercept was used to devise an equation for extrapolating other 2-weekly doses. C_max_ data was also extracted and used with C_min_ to calculate a range of C_diff_ values. A peak-to-trough ratio of 1.7 for 2-weekly dosing was assumed to determine C_max_ where this data was not available* (32)*. A further least-squares method using a zero intercept was used to devise an extrapolating equation for C_diff_ . Modelling using C_diff_ and a half-life calculator was then applied to determine C_min_ and C_max_ values for 3-weekly and 4-weekly dosing using C_diff_ data from the 2-weekly data.
Haloperidol – Pharmacokinetic data* (31) *was extracted for three 3-weekly dosages of 100mg, 200mg and 300mg. A least-squares regression method using a zero intercept was used to extrapolate values for 3-weekly C_min_ and C_diff_ . Modelling using C_diff_ and a half-life calculator was then applied to determine C_min_ and C_max_ values for 2-weekly and 4-weekly dosing using C_diff_ data from the 3-weekly data.
Zuclopenthixol – Pharmacokinetic data* (30) *was extracted for a range of 3-weekly dosages. A least-squares regression method using a zero intercept was used to extrapolate values for 3-weekly C_min_ and C_diff_ . Modelling using C_diff_ and a half-life calculator was then applied to determine C_min_ and C_max_ values for 2-weekly and 4-weekly dosing using C_diff_ data from the 3-weekly data.*

**Supplementary Table 2**

| **Drug** | **Half-life** | **t_max,_ (days)** | **Minimum effective dose** (47) | **C_max_ (ng/ml)** | **C_max_ D_2_ occupancy (%)** (41–43) |
| --- | --- | --- | --- | --- | --- |
| Flupentixol decanoate (33–35) | 17 (32) | 7 (32) | 50mg 4-weekly | 3.51 | 83.76 |
| Haloperidol decanoate (31) | 20 (31) | 7 (31) | 50mg 4-weekly | 1.63 | 76.17 |
| Zuclopenthixol decanoate (30) | 19 (29) | 7 (30) | 200mg 3-weekly | 8.26 | 87.70 |

*Pharmacokinetic and pharmacodynamic information for the three LIDAs modelled in our study at their ‘minimum effective dose’* (47)*.*

**Supplementary Figure 1**


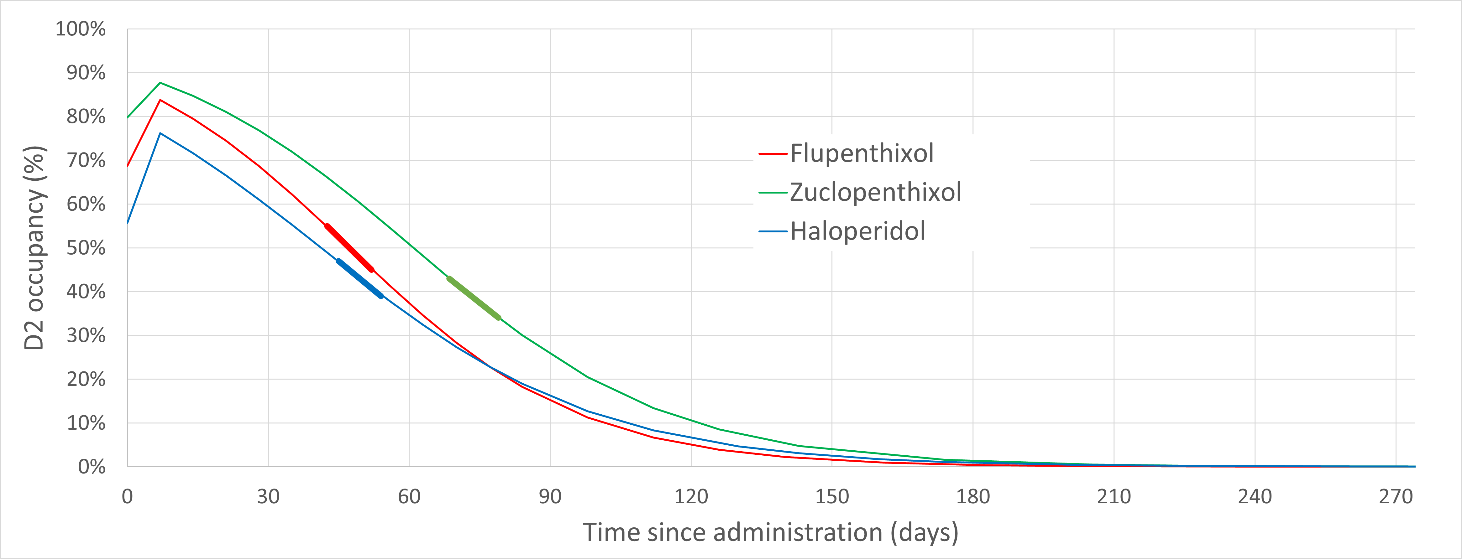

*Graph showing the change in D_2_* *occupancy resulting from abrupt discontinuation of three decanoate-based LIDAs from 'minimum effective dose’* (47). *Bold lines of corresponding colour demonstrate the peak RODOC for each LIDA.*

**Supplementary Table 3

a)**

| **Haloperidol dose & frequency** | **C_min_ (ng/ml)** | **D_2_ occupancy at C_min_ (%)** | **C_max_ (ng/ml)** | **D_2_ occupancy at C_max_ (%)** | **Absolute D_2_ occ. change during inter-dose interval (%)** |
| --- | --- | --- | --- | --- | --- |
| **300mg**  **3-weekly** | 6.85 | 90.97 | 12.8 | 94.96 | 3.99 |
| **200mg**  **3-weekly** | 4.57 | 87.04 | 8.54 | 92.62 | 5.58 |
| **100mg**  **3-weekly** | 2.28 | 77.06 | 4.27 | 86.26 | 9.2 |
| **300mg**  **2-weekly** | 16.22 | 95.98 | 22.18 | 97.03 | 1.05 |
| **300mg**  **4-weekly** | 3.83 | 84.92 | 9.78 | 93.5 | 8.58 |

*Tables showing modelled pharmacokinetics and respective D2 occupancies for various regimens of haloperidol decanoate.*

**b)**

| **Zuclopenthixol dose & frequency** | **C_min_ (ng/ml)** | **D_2_ occupancy at C_min_ (%)** | **C_max_ (ng/ml)** | **D_2_ occupancy at C_max_ (%)** | **Absolute D_2_ occ. change during inter-dose interval (%)** |
| --- | --- | --- | --- | --- | --- |
| **200mg**  **3-weekly** | 13.7 | 92.21 | 24.78 | 95.54 | 3.33 |
| **400mg**  **3-weekly** | 9.14 | 88.75 | 16.52 | 93.45 | 4.7 |
| **600mg**  **3-weekly** | 4.57 | 79.78 | 8.26 | 87.7 | 7.92 |
| **400mg**  **2-weekly** | 21.42 | 94.87 | 28.81 | 96.14 | 1.27 |
| **400mg**  **4-weekly** | 5.16 | 81.66 | 12.54 | 91.55 | 9.89 |

*Tables showing modelled pharmacokinetics and respective D2 occupancies for various regimens of zuclopenthixol decanoate.*

**Supplementary Table 4**

**a)**

|  | **Very Slow** | **Slow** | **Moderate** | **Fast** |
| --- | --- | --- | --- | --- |
| **Flupentixol** | 126mg 2-weekly  N/A 3-weekly  N/A 4-weekly | 57mg 2-weekly  205mg 3-weekly  N/A 4 weekly | 22mg 2-weekly  92mg 3-weekly  229mg 4-weekly | 6mg 2-weekly  34mg 3-weekly  97mg 4-weekly |
| **Haloperidol** | 186mg 2-weekly  N/A 3-weekly  N/A 4-weekly | 84mg 2-weekly  N/A 3-weekly  N/A 4-weekly | 33mg 2-weekly  172mg 3-weekly N/A 4-weekly | 9mg 2-weekly  66mg 3-weekly  184mg 4-weekly |
| **Zuclopenthixol** | 404mg 2-weekly  N/A 3-weekly  N/A 4-weekly | 182mg 2-weekly  N/A 3-weekly  N/A 4-weekly | 68mg 2-weekly  371mg 3-weekly N/A 4-weekly | 21mg 2-weekly  138mg 3-weekly  394mg 4-weekly |

*Thresholds for breaching regimen-specific D_2_ occupancy change for three LIDAs in terms of dose to the nearest milligram
N/A = no dosing below the licensed maximum at this interval will result in D_2_ occupancy change within the defined tapering threshold. This means it is not possible to taper LIDA at this frequency of administration in such a manner that will be consistent with gradual, hyperbolic tapering.*

**Supplementary Table 5**

|  | **Very Slow** | **Slow** | **Moderate** | **Fast** |
| --- | --- | --- | --- | --- |
| **Flupentixol** | 13.62 | 6.27 | 2.38 | 0.65 |
| **Haloperidol** | 10.06 | 5.68 | 2.43 | 1.08 |
| **Zuclopenthixol** | 21.63 | 16.07 | 7.5 | 3.21 |

*Thresholds for breaching regimen-specific D_2_ occupancy change for three LIDAs at 2-weekly intervals in terms of trough plasma drug concentration (C_min_, ng/ml)*
